# Vitamin B12 Status among Pregnant Women in the UK and Its Association with Obesity and Gestational Diabetes

**DOI:** 10.3390/nu8120768

**Published:** 2016-12-01

**Authors:** Nithya Sukumar, Hema Venkataraman, Sean Wilson, Ilona Goljan, Selvin Selvamoni, Vinod Patel, Ponnusamy Saravanan

**Affiliations:** 1 Warwick Medical School, University of Warwick, Coventry CV2 2DX, UK; N.Sukumar@warwick.ac.uk (N.S.); H.Venkataraman@warwick.ac.uk (H.V.); Vinod.Patel@warwick.ac.uk (V.P.); 2 Academic Department of Diabetes, Endocrinology and Metabolism, George Eliot Hospital, Nuneaton CV10 7DJ, UK; ilona.goljan@geh.nhs.uk (I.G.); selvamonis@geh.nhs.uk (S.S.); 3 Hull York Medical School, Hertford Building, University of Hull, Hull HU6 7RX, UK; hysdw3@hyms.ac.uk

**Keywords:** vitamin B12, gestational diabetes, obesity, macrosomia

## Abstract

Background: To evaluate vitamin B12 and folate status in pregnancy and their relationship with maternal obesity, gestational diabetes mellitus (GDM), and offspring birthweight. Methods: A retrospective case-control study of 344 women (143 GDM, 201 no-GDM) attending a district general hospital and that had B12 and folate levels measured in the early 3rd trimester was performed. Maternal history including early pregnancy body mass index (BMI) and neonatal data (birthweight, sex, and gestational age) was recorded for all subjects. Results: 26% of the cohort had B12 levels <150 pmol/L (32% vs. 22% in the two groups respectively, *p* < 0.05) while 1.5% were folate deficient. After adjusting for confounders, 1st trimester BMI was negatively associated with 3rd trimester B12 levels. Women with B12 insufficiency had higher odds of obesity and GDM (aOR (95% CI) 2.40 (1.31, 4.40), *p* = 0.004, and 2.59 (1.35, 4.98), *p* = 0.004, respectively), although the latter was partly mediated by BMI. In women without GDM, the lowest quartile of B12 and highest quartile of folate had significantly higher adjusted risk of fetal macrosomia (RR 5.3 (1.26, 21.91), *p* = 0.02 and 4.99 (1.15, 21.62), *p* = 0.03 respectively). Conclusion: This is the first study from the UK to show that maternal B12 levels are associated with BMI, risk of GDM, and additionally may have an independent effect on macrosomia. Due to the increasing burden of maternal obesity and GDM, longitudinal studies with B12 measurements in early pregnancy are needed to explore this link.

## 1. Introduction

The burden of maternal obesity (defined as body mass index (BMI) greater than 30 kg/m^2^) is rapidly increasing, affecting nearly 20% of pregnant women in the UK [1]. High BMI is associated with adverse pregnancy outcomes including recurrent miscarriages and maternal deaths [2]. In parallel, the incidence of gestational diabetes mellitus (GDM) has also risen affecting 5%–18% of all pregnancies depending on the diagnostic criteria applied [3,4].

Vitamin B12 (B12) and folate are essential micronutrients required for the synthesis of DNA, protein, and lipids, in a series of cellular reactions collectively known as one-carbon metabolism [5,6]. One step in this process is the conversion of homocysteine (Hcy) to a methyl donor, methionine, for which B12 and folate are necessary cofactors. Additionally, the mitochondrial conversion of methylmalonyl-CoA to succinyl-CoA requires B12 as a coenzyme and in its absence, accumulation of the former compound inhibits fatty acid oxidation, thereby promoting lipogenesis [7,8]. Therefore it can be postulated that low B12, at a cellular level, may be linked to adipocyte dysfunction and obesity-related complications by modulating lipid metabolism, cellular inflammation [9], and causing hypomethylation of cholesterol biosynthesis pathways [10].

A recent systematic review showed that B12 insufficiency among pregnant women across the world was common in all trimesters (20%–30%) [11]. Low B12 during pregnancy has implications for materno-fetal health including maternal adiposity, maternal and offspring insulin resistance [12,13,14], and adverse lipid profile in neonates [10,15]. The first two observations were replicated in a cohort of women without GDM from South West England [16] but there are no data available on the role of B12 in GDM in the UK.

Low B12 can have an impact on fetal birthweight by influencing placental development [17], although evidence for association with low birthweight (LBW) is equivocal [18,19,20]. At the other end of the spectrum, maternal obesity and insulin resistance are well-known to be associated with higher fetal birthweight [21,22]. Since B12 may be inversely associated with maternal BMI [12,16], it is possible that B12 is an independent mediator or a confounder for high birthweight.

The primary aim of our study is to investigate the B12 and folate status of pregnant women in the UK and their relationship with obesity and GDM, and secondarily to assess their relationship with fetal birthweight.

## 2. Methods

A retrospective case-control study of pregnant women attending the antenatal clinic in a district general hospital in the West Midlands, UK, between 2010 and 2013 was conducted. Using the hospital information database which had routine materno-fetal records of all deliveries during this period, we identified women who had a diagnosis of GDM and those who did not (labelled as no-GDM) and had their B12 levels measured in the 2nd or 3rd trimesters of their pregnancies. The no-GDM group consisted predominantly of women attending the medical obstetrics clinic for varying medical conditions. B12 and folate levels were measured routinely for screening for anaemia by the medical obstetric lead (VP), in addition to haemoglobin and ferritin. The physician (PS) running the antenatal-diabetes clinic measured these micronutrient levels for similar reasons in their first visit after the diagnosis of GDM. The following women were excluded from our analysis: pre-gestational diabetes (Type 1 and 2), multiple pregnancies, and those on vitamin B12 supplements at the time of blood sampling.

Clinical information about the women including medical and pregnancy history, smoking status, and BMI at booking was recorded from the database. Fetal outcomes such as birthweight, sex, and gestation were obtained for the secondary outcome analysis. Analysis of glucose was done by a hexokinase enzymatic method in the hospital laboratory and serum B12 and folate by an electrochemiluminescent immunoassay using a Roche Cobas immunoassay analyser (Roche Diagnostics UK, Burgess Hill, UK).

### 2.1. Definitions

A selective screening approach was used to screen high-risk women for GDM according to the National Institute for Health and Care Excellence (NICE) guidelines [23] (i.e., BMI > 30 kg/m^2^, previous GDM, previous macrosomia, first degree relative with diabetes, and ethnic minority race). This consisted of a 2-h 75 g glucose tolerance test (GTT) between 26 and 28 weeks of gestation. The modified World Health Organisation (WHO) 1999 criteria was used to diagnose GDM (fasting glucose ≥ 6.1 mmol/L or 2-h glucose ≥ 7.8 mmol/L) during the study period. The reference range for serum B12 was 150–489 pmol/L and for serum folate was 7.0–42.4 nmol/L, respectively. Insufficiency of the two micronutrients were defined as <150 pmol/L and <7 nmol/L, respectively [12,16]. Birthweight percentiles and *z*-scores were calculated using gestational age at delivery and sex-specific reference standards published by the Intergrowth calculator 21st Project [24]. Macrosomia was defined as birthweight > 4000 g, large for gestational age (LGA) as >90th percentile for sex and gestational age, LBW as <2500 g, and small for gestational age (SGA) as <10th percentile for sex and gestational age.

### 2.2. Statistical Analysis

Based on the pilot data, the required sample size in each group to demonstrate a 15% difference in mean B12 with 90% power and at 5% significance was calculated to be 144. Statistical analysis was performed using SPSS version 22.0 [25]. Since BMI, serum B12, and folate were not normally distributed, they were log-transformed for statistical purposes. For comparison of GDM and no-GDM mothers, the Student’s *t*-test was used for continuous variables (e.g., B12, folate, and BMI) and the Chi-square test for categorical variables. Stepwise multiple linear regression was performed with B12 and folate as the dependent variables with the predictors entered or removed from the model according to the following criteria: Probability-of-*F*-to-enter ≤ 0.050, Probability-of-*F*-to-remove ≥ 0.100. Logistic regression was performed to determine the odds of maternal obesity and GDM according to B12/folate insufficiency status and the risk of macrosomia, LGA, LBW, and SGA according to quartiles of B12/folate. The regression models included the following co-variates: age, parity, ethnic origin, smoking, gestation of bloods, BMI, B12, and folate (where appropriate). For macrosomia and LBW, sex and gestational age were additionally added to the models.

Our institution has obtained ethics approval to collect B12 and folate data from pregnant women in an anonymised form (NHS ethics committee reference number 12/LO/0239).

## 3. Results

Out of approximately 8400 deliveries in the hospital between 2010 and 2013 that were screened, 344 women (143 GDM, 201 no-GDM) who met the inclusion criteria and had B12 levels measured in the 3rd trimester of pregnancy were included. The clinical characteristics of the whole cohort and by GDM status are provided in Table 1. Of the 201 no-GDM women, 45% had GTT as per NICE selective screening criteria [23] and the characteristics of these women are summarised in the Supplementary Materials Table S1.

For the whole cohort, the mean gestation of serum vitamin B12 and folate measurements was at 26.9 weeks and GTT was at 26.6 weeks. B12 levels were lower in women with GDM (169.0 vs. 195.6 pmol/L, *p* < 0.001) and a significantly higher proportion of women with GDM had B12 insufficiency compared to non-GDM (Table 1). Folate deficiency was rare and 91% of the whole cohort was taking folate supplements. Serum folate levels were not different in the two groups.

### 3.1. Vitamin B12, Folate Status, Maternal BMI, and GDM

**Figure 1:**
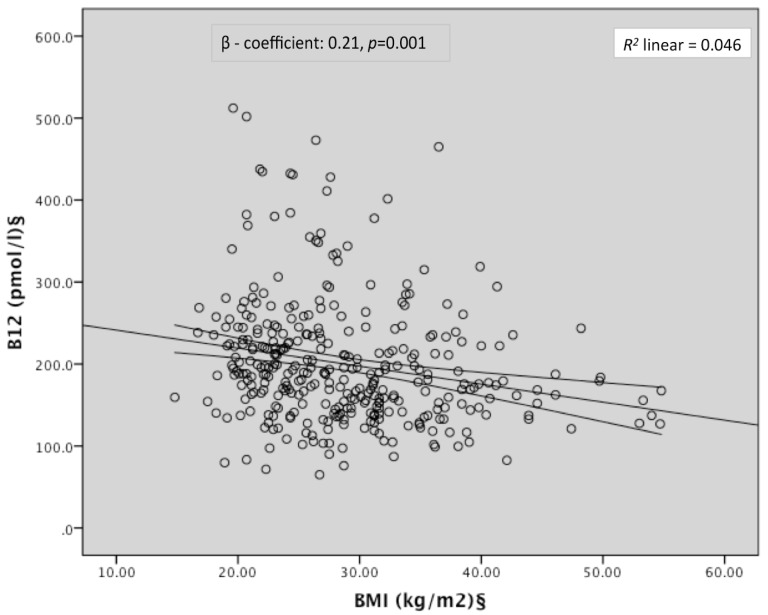
Scatterplot of the correlation between BMI and serum B12. § Log-transformed for statistical comparisons; Regression model included age, parity, ethnicity, smoking, gestation of bloods, folic acid supplements, and serum folate.

Women with B12 insufficiency had higher 1st trimester BMI than those without (30.9 ± 7.56 vs. 28.0 ± 7.30 kg/m^2^, *p* < 0.05). After adjusting for age, parity, ethnicity, smoking status, and gestation of blood tests, BMI was a significant negative predictor of B12 (β coefficient −0.21; 95% CI: −0.47, −0.13; *p* = 0.001) whilst serum folate showed a positive association with B12 (Table 2, Figure 1). BMI was also negatively associated with serum folate after adjustment although the strength of association was weaker (β coefficient −0.12; 95% CI: 0.00, 0.33; *p* = 0.05). Third trimester vitamin B12 insufficiency was additionally associated with a 2.4 times higher odds of first trimester obesity (Table 3).

B12 deficient women were at 2.59-times higher odds of having a diagnosis of GDM after adjusting for age, parity, ethnic origin, smoking, gestation of bloods, and serum folate (Table 3). The effect size was weaker when maternal BMI was added into the model (aOR 2.05, *p* = 0.04). Folate deficiency was not significantly associated with a risk of GDM. There was also no association seen between folate thresholds and obesity.

### 3.2. Vitamin B12, Folate, and Birth Outcomes

Birth outcome data were available in 335 women (97% of total cohort) and one baby born at less than 32 weeks gestation was excluded from this analysis. 54.5% of the babies were male and the mean birthweight was 3353 g. GDM women delivered 10 days earlier than no-GDM women and their mean offspring birthweight was 180 g lower (3250 vs. 3428 g, *p* < 0.01) (Supplementary Materials Table S2). Due to the likely confounding effects of treatment in GDM women, the relationship between maternal B12 and folate and birth outcomes were analysed only in no-GDM women (Table 4). Women in the lowest quartile of B12 had higher rates of macrosomic babies compared to the highest quartile (22.9% vs. 8.0%) (Table 4). After adjustment for age, parity, ethnicity, smoking, serum folate, gestation of B12 bloods, and newborn sex and gestational age, the relative risk (RR) of fetal macrosomia was higher in women in the lowest quartile (RR 5.26, 95% CI: 1.26, 21.91, *p* = 0.02). The significance was attenuated when gestational BMI was added to the model (Table 4). A similar trend for the risk of LGA was observed although the result did not reach statistical significance. There was no association between B12 thresholds and the outcomes of LBW or SGA. The impact of serum folate on fetal macrosomia showed the reverse pattern for all of these outcome measures. Women in the highest quartile of folate had significantly higher risk of fetal macrosomia compared to those in the lowest quartile (RR 4.99, 95% CI: 1.15, 21.62, *p* = 0.03), which remained significant after adjusting for maternal BMI (RR 6.60, 95% CI: 1.42, 30.71, *p* = 0.02) (Table 4).

## 4. Discussion

Our study, although retrospective in nature, showed three key findings. Firstly, it is the first study to show that low B12 status in pregnancy is associated with a higher risk of GDM in a UK population. Secondly, higher first trimester BMI was an independent predictor of later B12 insufficiency. Thirdly, low B12 levels were associated with macrosomia in the subgroup of no-GDM women, which seems to be partly mediated by maternal BMI.

The only other study that examined the link between B12 and GDM by Krishnaveni et al. was in an Indian cohort [12]. The magnitude of association found in that study was similar to ours, but the significance was lost after adjusting for maternal BMI. In our study, although the effect size was reduced when adjusted for BMI (aOR 2.59 vs. 2.05; Table 3), the significance persisted, suggesting a potential independent effect of B12. Higher numbers of women with GDM in our cohort and a ‘case-control’ design might explain the larger effect size. The recent finding by Knight et al., albeit in no-GDM women, also supports the inverse link between B12 levels and insulin resistance in pregnant White Caucasian women [16]. Indeed, higher insulin resistance in the context of low B12 has been shown by other authors in obese adolescents [26], non-pregnant adults [27,28], as well as in women with polycystic ovarian syndrome [28]. Prospective longitudinal studies are needed to investigate whether the presence of low B12 status in early pregnancy independently increases the risk of incident GDM.

The aetiology of the inverse relationship between B12 and BMI found in our study is an intriguing one. While confounding factors such as dietary habits, socioeconomic status, and hemodilution may be present, other studies that have corrected for these still show an independent link between B12 and BMI [12,16]. Interestingly, the frying and roasting of meat products reduces the bioavailability of B12 by 20%–40% [29], so higher consumption of processed foods may increase the risk of both B12 insufficiency and metabolic diseases. Additionally, B12 has been shown to be negatively associated with other markers of obesity such as triglycerides [7], blood pressure [30], and the metabolic syndrome [31], which lends support to a possible pathological association between them. In one trial, the supplementation of B12 and folate in adults with metabolic syndrome improved insulin resistance by ameliorating endothelial dysfunction, providing further insight into how these conditions may be linked [32]. Further studies are needed to determine the direction of association and a potential reverse causality.

This is the first study that has demonstrated a relationship between maternal B12 and macrosomia, which seem to be mediated in part by maternal obesity. We demonstrated this only in no-GDM women as the treatment of GDM is a major confounder for macrosomia. Unfortunately, we did not have adiposity measures or a bigger sample size to assess the interactions between B12 status and maternal BMI/adiposity with offspring size and adiposity.

The rates of B12 insufficiency observed in our no-GDM population was similar to that observed by Knight et al. [16] (22% vs. 20%), suggesting that such higher rates of insufficiency are not limited to Indian populations [11]. It must be noted that a fall in B12 during pregnancy may be physiological due to a decrease in the fraction bound to inactive haptocorrin [33], but the evidence is equivocal with regards to whether there is also a fall in the active form, holotranscobalamin [34,35]. In the absence of specific cut-off values to define B12 deficiency in pregnancy, we used the non-pregnant reference range (<150 pmmol/L). It is noteworthy that associations with adverse maternal metabolic outcomes [12] and elevation in Hcy during pregnancy [36] were found by other authors at B12 thresholds similar to this.

It was reassuring to see that folate deficiency was rare, albeit in this selected hospital-based cohort. However, the combination of low B12 and high folate has been shown to be associated with lower neonatal birthweight [37] as well as central adiposity and insulin resistance in 6-year old offspring [14]. Whilst our sample size was not large enough to perform a detailed subgroup analysis, we observed that women in the lowest quartile of B12 and highest quartile of folate had similar risks of macrosomia (aRR of 5.3 and 4.99; Table 4). Therefore, it is possible that the women with such a B12-folate imbalance are particularly at high risk of having larger babies. This phenomenon (high folate/low B12), is increasingly common in populations with mandatory folic acid fortification such as in the USA and Canada [38,39], and is related to adverse clinical outcomes in the elderly population [40].

Although we have identified associations between B12, maternal obesity, risk of GDM, and fetal macrosomia, our study does not prove causation or the direction of the relationship between these factors. Some of the important limitations were that this was a single-centre, retrospective study involving pregnant women attending a hospital clinic. Therefore it was not possible to obtain early pregnancy B12/folate levels. We adjusted for the gestation of bloods in all the regression analyses, to reduce some of the bias due to longitudinal changes in B12 during pregnancy. We did not have markers of adiposity, and therefore it was not possible to study the potential differential association of low B12 status with obesity and adiposity in pregnant women as well as their offspring. Lack of functional measures of B12 insufficiency, such as Hcy and methylmalonic acid (MMA), or holotranscobalamin, which is the active fraction of B12 available for uptake by tissues, limits the ability to study the thresholds of B12 sufficiency during pregnancy and should be measured in future studies.

## 5. Conclusions

We have shown for the first time in a UK population that B12 deficiency in pregnancy is common particularly in obese women, is independently associated with GDM, and may contribute to macrosomia. As the prevalence of maternal obesity and GDM is rapidly increasing, our findings warrant longitudinal cohort studies to understand the interplay between B12 and these outcomes. If early pregnancy B12 status is found to be independently predictive of incident GDM, such findings could potentially offer simple interventions to improve the metabolic health of pregnant women and their offspring.

## Figures and Tables

**Table 1:** Maternal characteristics according to GDM status.

Variables	Total	GDM	No GDM
Number (%)	344 (100)	143 (41.6)	201 (58.4)
Age (years)	30.3 ± 5.88	31.4 + 5.8	29.6 ± 5.9 **^,a^
BMI (kg/m^2^) §	28.8 ± 7.46	31.7 ± 7.0	26.7 ± 7.1 ***
Obesity (BMI > 30 kg/m^2^) (%)	38.0	60.6	22.0 ***
Current smokers (%)	18.7	15.2	19.9
Parity	1.1 ± 1.18	1.2 ± 1.18	1.0 ± 1.18
Ethnicity (%)			
European	86.9	86.0	87.6
South Asian	9.3	11.2	8.0
Afro-Caribbean	1.2	0.7	1.5
Other	1.2	1.4	1.0
Gestation of GTT (weeks) ^b^	26.6 ± 3.95	26.4 ± 4.40	26.8 ± 3.10
Mean fasting glucose (mmol/L) §	4.9 ± 1.01	5.2 ± 1.15	4.4 ± 0.39 ***
Mean 2 h glucose (mmol/L) §	7.5 ± 1.94	8.7 ± 1.26	5.6 ± 1.13 ***
Gestation of B12 bloods (weeks)	26.9 ± 5.3	28.0 ± 4.3	26.2 + 5.7 **
Vitamin B12 (pmol/L) §	187.5 (146.9, 235.4)	169.0 (140.2, 217.7)	195.6 (157.9, 244.6) **
Vitamin B12 deficiency (<150 pmol/L), *n* (%)	90 (26.2)	46 (32.2)	44 (21.9) *
Serum folate (nmol/L) §	21.3 (14.0, 34.4)	21.5 (13.5, 34.5)	20.8 (14.5, 34.4)
Serum folate deficiency (<7 nmol/L), *n* (%)	5 (1.5)	3 (2.1)	2 (1.0)
Folic acid supplements taken (%)	91.4	90.9	91.5

Continuous variables are mean ± SD (or median (IQR)), categorical variables are percentages; ^a^ *p*-value as compared to the GDM group, * *p* < 0.05, ** *p* < 0.01, *** *p* < 0.001; ^b^ GTT results available in 90/201 (44.8%) of no-GDM women; § Log-transformed for statistical comparison, GDM: gestational diabetes mellitus, BMI: body mass index, GTT: glucose tolerance test.

**Table 2:** Predictors of vitamin B12 and folate.

Variables	Serum B12 §		Serum Folate §	
	β-Coefficient	*p*-Value	β-Coefficient	*p*-Value
Age	-	NS	0.32	<0.001
Parity	-	NS	−0.24	<0.001
BMI §	−0.21	0.001	−0.12	0.05
Ethnicity	-	NS	-	NS
Smoking	-	NS	-	NS
Gestation of B12/folate bloods	-	NS	−0.28	<0.001
Serum B12 §			0.12	0.05
Serum folate §	0.23	<0.001		
Folic acid supplements	-	NS	-	NS

§ Log-transformed for statistical calculations; NS: non-significant.

**Table 3:** Relationship of maternal B12 and folate with obesity and gestational diabetes.

	*n* (%)	Obesity, *n* (%)	GDM, *n* (%)
Vitamin B12 deficiency			
Yes	90	44 (49.4)	46 (51.1)
No	254	86 (34.0)	97 (38.2)
Model 1 OR (95% CI) ^a^		2.40 (1.31, 4.40)	2.59 (1.35, 4.98)
adjusted *p*		0.004	0.004
Model 2 OR (95% CI) ^b^		N/A	2.05 (1.03, 4.10)
adjusted *p*		N/A	0.042
Folate deficiency			
Yes	5	4 (80.0)	3 (60.0)
No	332	125 (37.9)	139 (41.9)
Model 1 OR (95% CI) ^a^		6.29 (0.48, 82.79)	1.93 (0.17, 22.23)
adjusted *p*		NS	NS
Model 2 OR (95% CI) ^b^		N/A	0.89 (0.07, 11.38)
adjusted *p*		N/A	NS

Table showing the proportions and odds ratio of obesity and development of GDM according to the thresholds of B12 and folate (reference categories are ‘No B12/folate deficiency’); ^a^ Model 1 adjusted for age, parity, ethnic origin, smoking, gestation of bloods, and serum folate (or B12, respectively); ^b^ as for Model 1 plus gestational BMI; N/A: not applicable; NS: non-significant.

**Table 4:** Relationship between maternal B12 on birth outcome measures in no-GDM women.

	*n*	Range of Values (pmol/L)	Macrosomia, *n* (%)	LGA, *n* (%)	LBW, *n* (%)	SGA, *n* (%)
Vitamin B12 (quartiles)						
1	48	71.6, 157.2	11 (22.9)	12 (25.0)	1 (2.1)	4 (8.3)
2	48	158.7, 195.6	10 (20.8)	12 (25.0)	2 (4.2)	2 (4.2)
3	47	196.3, 244.3	9 (19.1)	10 (21.3)	3 (6.4)	3 (6.4)
4	50	245.0, 512.2	4 (8.0)	5 (10.0)	3 (6.0)	5 (10.0)
Relative risk (95% CI) ^a^			5.26 (1.26, 21.91)	3.18 (0.96, 10.56)	0.10 (0.002, 5.75)	1.35 (0.28, 6.47)
*p* ^b^			0.02	0.06	0.27	0.71
*p* ^c^			0.05	0.13	0.37	0.52
Folate (quartiles)						
1	44	4.5, 14.3	5 (11.4)	7 (15.9)	4 (9.1)	4 (9.1)
2	47	14.5, 20.6	7 (14.9)	9 (19.1)	1 (2.1)	2 (4.3)
3	48	20.8, 34.2	11 (22.9)	10 (20.8)	1 (2.1)	3 (6.3)
4	48	34.4, 45.3	10 (20.8)	12 (25.0)	3 (6.3)	5 (10.4)
Relative risk (95% CI) ^a^			4.99 (1.15, 21.62)	2.32 (0.74, 7.34)	0.21 (0.01, 9.64)	1.52 (0.26, 8.93)
*p* ^b^			0.03	0.15	0.42	0.64
*p* ^c^			0.02	0.06	0.41	0.90

^a^ Relative risk of birthweight outcome in quartile 1 vs. quartile 4 of B12 and quartile 4 vs. quartile 1 of folate; ^b^ adjusted for age, parity, ethnic origin, smoking, gestation of bloods, and serum folate (or B12, respectively), plus sex and gestational age for macrosomia and LBW; ^c^ as for Model b plus gestational BMI; LGA: large for gestational age; LBW: low birthweight; SGA: small for gestational age.

## Supplementary Materials

The following are available online at http://www.mdpi.com/2072-6643/8/12/768/s1, Table S1: Table of characteristics of no-GDM women who did and did not undergo GTT, Table S2: Table of birth outcomes of offspring according to maternal GDM status.
