# Supplementary material for: Vitamin B12 Status among Pregnant Women in the UK and Its Association with Obesity and Gestational Diabetes
Source: Nutrients. 2016 Dec 1;8(12):768. doi: 10.3390/nu8120768 (PMC5188423; doi:10.3390/nu8120768)
Supplement: Supplementary file 1 [file nutrients-08-00768-s001.docx]

Supplementary Materials: Vitamin B12 Status among Pregnant Women in the UK and Its Association with Obesity and Gestational Diabetes

Nithya Sukumar, Hema Venkataraman, Sean Wilson, Ilona Goljan, Selvin Selvamoni, Vinod Patel and Ponnusamy Saravanan

**Table S1.** Table of the characteristics of no-GDM women who did and did not undergo GTT.

| **Variables** | **GTT** | **No GTT** |
| --- | --- | --- |
| Number (%) | 90 (44.8) | 111 (55.2) |
| Age (years) | 30.2 ± 5.9 | 29.1 ± 5.8 |
| BMI (kg/m^2^) § | 29.7 ± 8.5 | 24.3 ± 4.5 ***^,a^ |
| Obesity (%) | 43.3 | 4.5 *** |
| Current smokers (%) | 21.1 | 18.9 |
| Parity | 1.0 ± 1.1 | 1.1 + 1.2 |
| Ethnicity (%) |  |  |
| European | 87.8 | 87.4 |
| South Asian | 8.9 | 7.2 |
| Afro-Caribbean | 1.1 | 1.8 |
| Other | 1.1 | 0.9 |
| Gestation of B12 bloods (weeks) | 26.5 ± 5.7 | 25.9 ± 5.8 |
| Vitamin B12 (pmol/L) § | 180.4 (146.7, 221.6) | 218.4 (163.1, 259.8) * |
| Vitamin B12 deficiency (<150 pmol/L) (%) | 25.6 | 18.9 |
| Serum folate (nmol/L) § | 22.0 (14.5, 34.9) | 19.7 (14.3, 34.0) |
| Serum folate deficiency (<7 nmol/L) (%) | 1.1 | 0.9 |

Continuous variables are mean ± SD (or median (IQR)), categorical variables are percentages. ^a^ *p*-value as compared to undergone-GTT group, * *p* < 0.05, *** *p* < 0.001. § Log-transformed for statistical comparison; GTT: glucose tolerance test; BMI: body mass index.

**Table S2.** Table of the birth outcomes of offspring according to maternal GDM status.

| **Variables** | **Total** | **GDM** | **No GDM** |
| --- | --- | --- | --- |
| Number (%) | 334 (100) | 141 (42.2) | 193 (57.8) |
| Mean fasting glucose (mmol/L) § | 4.9 ± 1.01 | 5.2 ± 1.15 | 4.4 ± 0.39 ***^,a^ |
| Mean 2hr glucose (mmol/L) § | 7.5 ± 1.94 | 8.7 ± 1.26 | 5.6 ± 1.13 *** |
| Birthweight (g) | 3353 ± 528 | 3250 ± 411 | 3428 ± 588 ** |
| Sex, *n* (%) male | 182 (54.5) | 76 (53.9) | 106 (54.9) |
| Gestation at birth (days) | 274 ± 10.7 | 268 ± 6.4 | 278 ± 11.3 *** |
| Macrosomia, *n* (%) | 42 (12.6) | 8 (5.7) | 34 (17.6) ** |
| LGA, *n* (%) | 68 (20.4) | 29 (20.6) | 39 (20.2) |
| Low birthweight, *n* (%) | 14 (4.2) | 5 (3.5) | 9 (4.7) |
| SGA, *n* (%) | 19 (5.7) | 5 (3.5) | 14 (7.3) |

Continuous variables are mean ± SD (or median (IQR)), categorical variables are percentages. ^a^ *p*-value as compared to GDM group, ** *p* < 0.01, *** *p* < 0.001. § Log-transformed for statistical comparison; GDM: gestational diabetes mellitus; LGA: large for gestational age; SGA: small for gestational age.
